# The inflammasome: an emerging therapeutic oncotarget for cancer prevention

**DOI:** 10.18632/oncotarget.9391

**Published:** 2016-05-17

**Authors:** Wang Zhiyu, Neng Wang, Qi Wang, Cheng Peng, Jin Zhang, Pengxi Liu, Aihua Ou, Shaowen Zhong, Mario D. Cordero, Yi Lin

**Affiliations:** ^1^ Department of Mammary Disease, Guangdong Provincial Hospital of Chinese Medicine, Guangzhou, China; ^2^ Department of Breast Oncology, Sun Yat-sen Univeristy Cancer Center, State Key Laboratory of Oncology in South China, Collaborative Innovation Center for Cancer Medicine, Guangzhou, Guangdong, China; ^3^ Institute of Clinical Pharmacology, Guangzhou, China; ^4^ Pharmacy College, State Key Laboratory Breeding Base of Systematic Research, Development and Utilization of Chinese Medicine Resources, Chengdu University of Traditional Chinese Medicine, Chengdu, China; ^5^ College of Basic Medicine, Guangzhou University of Chinese Medicine, Guangzhou, China; ^6^ Research Laboratory, Oral Medicine Department, University of Seville, Seville, Spain

**Keywords:** inflammasome, NOD-like receptors, inflammation, cancer prevention, IL-1β/IL-18

## Abstract

Deregulated inflammation is considered to be one of the hallmarks of cancer initiation and development regulation. Emerging evidence indicates that the inflammasome plays a central role in regulating immune cells and cytokines related to cancer. The inflammasome is a multimeric complex consisting of NOD-like receptors (NLRs) and responds to a variety of endogenous (damage-associated molecular patterns) and exogenous (pathogen-associated molecular patterns) stimuli. Several lines of evidence suggests that in cancer the inflammasome is positively associated with characteristics such as elevated levels of IL-1β and IL-18, activation of NF-κB signaling, enhanced mitochondrial oxidative stress, and activation of autophagic process. A number of NLRs, such as NLRP3 and NLRC4 are also highlighted in carcinogenesis and closely correlate to chemoresponse and prognosis. Although conflicting evidence suggested the duplex role of inflammasome in cancer development, the phenomenon might be attributed to NLRs difference, cell and tissue type, cancer stage, and specific experimental conditions. Given the promising role of inflammasome in mediating cancer development, precise elucidation of its signaling network and pathological significance may lead to novel therapeutic options for malignancy therapy and prevention.

## INTRODUCTION

Inflammation is recognized as a major hallmark of cancer. As early as 1863, Rudolph Virchow speculated on a link between cancer and inflammation based on the observation of leukocyte infiltration in human breast cancer [1, 2]. It is generally accepted that up to 25% of malignancies are related to chronic inflammation, chronic infection, or both [3-5]. Numerous studies provide evidence that chronic inflammation facilitates resistance to growth inhibition, independent neoangiogenesis, apoptotic evasion, malignant transformation, and metastatic potential obtainment [6]. During tumor initiation, oxidative molecules including reactive oxygen species and reactive nitrogen species induced by tumor infiltrating immune cells induce epigenetic alterations in oncogenes or tumor suppressive genes, thereby promoting carcinogenesis [7-9]. On the other hand, during tumor progression and metastasis, cytokines or chemokines secreted by immune cells lead to an increase in cell survival, motility and invasiveness, such as epithelial-mesenchymal transition (EMT) [10-12]. Elucidating the molecular network between inflammation and cancer risk is of great significance for cancer prevention and treatment.

Once invaded by harmful microbes or foreign particles, germline-encoded pattern recognition receptors (PRRs) constitute the first line of defense. The PRR superfamily includes members of the Toll-like receptors (TLR), nucleotide-binding and oligomerization domain containing receptors (NOD-like receptors, NLRs), retinoic acid-inducible gene (RIG) I-like RNA helicases, C-type lectins, and AIMs like receptors (ALR) [13-15]. The molecular targets of PRRs usually include pathogen associated molecular patterns (PAMPs) and danger associated molecular patterns (DAMPs). Binding of PAMPs or DAMPs to these receptors leads to an initiation of the host's immune response by activation of inflammatory cells and a number of transcription factors such as NF-κB, STAT, and FOXO [16, 17]. The multimeric inflammasome complex senses all these processes.

Jurg Tschopp was the first to identify the inflammasome in 2002 [18]. Its structure consists of an assembly, either of the NLR proteins, NLRP1, NLRP3, NLRC4, NLRP6, and NAIP5 or the DNA-sensing complex of AIM2, a member of the interferon-inducible HIN-200 protein family [19]. Activation of inflammasome leads to NLR oligomerization and subsequent interaction with the adaptor protein ASC and the CARD domain of caspase-1. Caspase-1 in turn, regulates the maturation of proinflammatory cytokines interleukin-1β (IL-1β) and IL-18 or the rapid inflammatory form of cell death called pyroptosis [20-22]. Notably, the level of IL-1β and IL-18 were found to be significantly elevated in various types of malignancies. These cytokines can facilitate pro-carcinogenic activity by triggering the secretion of VEGF, FGF2 and STAT3, and subsequently support cancer survival and distant metastasis [23-25]. Therefore, elucidating the molecular network of inflammasomes has become a novel strategy for cancer prevention research.

## INFLAMMASOME CASCADE SIGNALING

**Figure 1:**
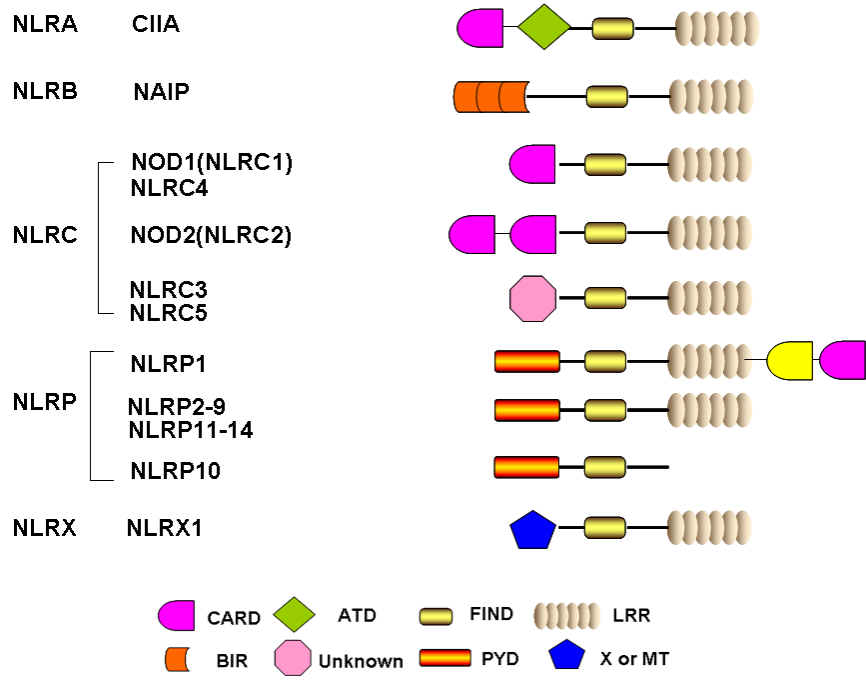
Schematic representation of the basic structure of individual NLR domain Human NLRs were classified into five categories including NLRA, NLRB, NLRC, NLRP and NLRX. All 22 human NLRs contain a central NACHT domain and a C-terminal ligand sensing domain LRR, with the exception of NLRP10. The N-terminal domain of each NLR is specific and responsible for ascribing different biofunctions. CARD: caspase association and recruitment domain; ATD: acidic transactivation domain; FIND: function to find domain; PYD: pyrin domain; BIR: Baculoviral inhibition of apoptosis protein repeat domain; LRR: leucine-rich repeats; MT: targets NLRX1 to the mitochondria but no sequence homology with tranditional mitochondrial targeting sequence has been reported.

**Figure 2:**
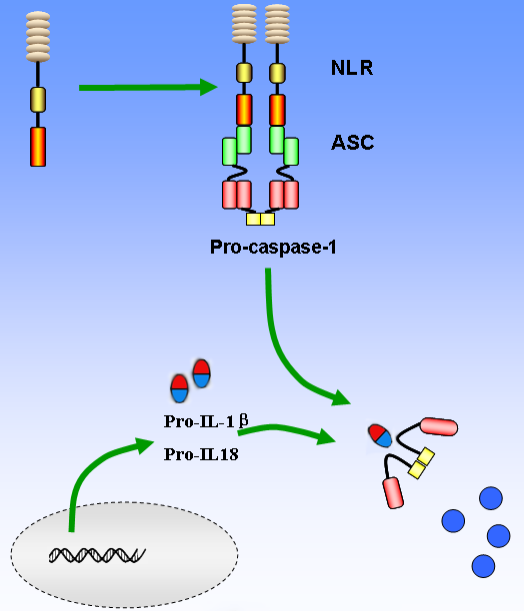
Basic mechanisms of activation of the main NLRs inflammasome The recognition of PAMPs and/or DAMPs leads to NOD domain oligomerization, which in turn facilitates recruitment of pro-caspase-1 *via* the CARD domain interactions between pro-caspase-1 and adaptor protein ASC. Pro-caspase-1 will be then cleaved and converts pro- IL-1βand pro-IL-18 into their active forms to amplify the inflammatory response. On the other hand, caspase-1 can lead to cell pyropotosis with the consequence of membrane rupture and release of alarmins such as IL-1α and HMGB1.

Compared to TLRs that are usually located on the membrane, NLRs are intracellular molecules and classified into 22 and 34 isoforms in human and mouse genome, respectively. The NLRs are characterized by a tripartite structure, consisting of a carboxy-terminal leucine-rich repeat domain, a central nucleotide-binding oligomerization domain, and a variable N-terminal protein-protein interaction domain, which can be either a Pyrin domain (PYD), a caspase recruitment and activation domain (CARD), or a baculovirus inhibitor of apoptosis repeat domain (BIR) (Figure 1) [26, 27]. The common NLRs and their functions and ligands are summarized in Table 1. The leucine-rich repeat domain appears to act as a ligand sensing component of NLRs; however, the molecular basis of ligand-binding mechanisms of NLRs is poorly understood [28]. The nucleotide-binding oligomerization domain facilitates recruitment of pro-caspase-1 via interactions between pro-caspase-1 and adaptor protein ASC, which take place in the CARD domain [29]. The PYD domain of ASC interacts with NLRs and its CARD domain binds directly with pro-caspase-1. Once pro-caspase-1 is recruited to the inflammasome, it will be cleaved into a p35 and p10 fragments in a proximity-induced multimerization manner. The p35 fragment will subsequently be processed into the CARD and a p20 subunit. The p10 fragment together with 2 molecules of p20 will finally form an active caspase-1 enzyme, which converts pro- IL-1βand pro-IL-18 into their active forms (Figure 2) [30, 31]. Furthermore, pyroptosis could also be induced following caspase-1 activation and its activation is considered to be a critical mechanism fighting against Gram-negative and Gram-positive bacteria [32].

**Table 1:** Inflammasome and non-inflammasome forming NLRs, functions and their ligands

NLRs	NLR family	functions	Ligands
NLRP3	NLRP	Interacts with caspase-1 and ASC; activates NF-κB signaling and IL-1β /IL-18 release	Muramyl dipeptide, LPS, Bacterial and viral DNA/RNA, silica, amyloid-β fibrils, extracellular ATP
NLRC4	LRC	Interacts with caspase-1, ASC and NAIP; elevates NF-κB signaling and IL-1β /IL-18 release	Flagellin from *Salmonella, Legionella, Listeria, Pseudomonas*
NAIP	NLRB	Formation of NAIP/NLRC4 inflammasome complex	Flagellin from Legionella
NLRP6	NLRP	Inflammasome complex formation with ASC and caspase-1; activates NF-κB signaling and IL-1β /IL-18 release	Ligands unknown
NLRP1	NLRP	Inflammasome complex formation with ASC and caspase-1	Muramyl dipeptide, *Toxoplasma gondii* and *Bacillus anthracis* lethal toxin
NLRP12	NLRP	Inhibits IRAK1, TRAF3 and NIK, attenuates both canonical and non-canonical NF-κB signaling	Ligands unknown
NLRX1	NLRX	Inhibits TRAF6 and attenuates canonical NF-κB signaling	Viral RNA
NLRC3	NLRC	Inhibits TRAF6 and attenuates canonical NF-κB signaling	Ligands unknown
NOD1	NLRC	Interacts with RIP2 and recruits RICK and CARD9	GM-tripeptide γ-d-Glu-DAP(iEDAP) d-lactyl-l-Ala-γ-Glu-meso-DAP-Gly (FK156) heptanolyl-γ-Glu-meso-DAP-Ala
NOD2	NLRC	Recruits RIP2 and activates NF-κB and MAPK pathways; negatively regulated by CARD8	Muramyl dipeptide MurNAc-l-Ala-g-d-Glu-l-Lys (M-TRlys)

Alternatively, inflammasome could also be activated through a non-canonical pathway, which involves caspase-11 or caspase-8. Caspase-11 was found to be necessary for the maturation of IL-1β and IL-18 in enteric bacteria such as *Escherichia coli, Citrobacter rodentium, and Vibrio cholera*. After recruitment to the inflammasome, pro-caspase-11 is cleaved into the p26 subunit and subsequently interacts with caspase-1 [33]. Studies show that caspase-8 was necessary for the inflammasome activation in LPS-primed macrophages and dendritic cells [34]; however, the detailed interaction mode and mechanisms are poorly understood.

## REGULATION OF INFLAMMASOME ACTIVATION

Given that IL-1β, IL-18 and pyroptotic death response have the potential to damage the host, tight control of inflammasome activation is of great significance for the prevention of disease progression. According to the 2-signal model of inflammasome induction, NF-κB is thought to serve as the first signal that primes NLR and pro-IL-1β expression [17, 22, 35]. Constitutive activation of NF-κB is shown in a wide variety of tumor types, such as lymphoma, liver cancer, lung cancer, breast cancer, etc [36, 37]. Besides, NF-κB is activated in response to carcinogenic processes such as tobacco, stress, obesity, alcohol, infectious agents, irradiation, etc [36, 37]. Furthermore, NF-κB controls the expression of the genes linked with proliferation, invasion, angiogenesis, and metastasis of cancer [38]. Besides, NF-κB activation further upregulates a series of inflammatory factors, such as TNFα, IL-6, IL-1 and IL-8, which constitute a positive feedback loop to induce cellular and DNA damage and to promote cell proliferation and transformation [36]. A previous study also demonstrated that NLRP3 promoter contains putative NF-κB binding site and NF-κB inhibition resulted in a significant reduction of NLRP3 expression [39]. Meanwhile, mounting evidence suggested that NLRP3 inflammasome formation is positively associated with NF-κB activity following drug treatment such as LPS, CPT-11, FGF-21, etc [40-42]. All these findings implied that the pro-tumorigenic ability of NF-κB might be attributed to inflammasome activation.

**Figure 3:**
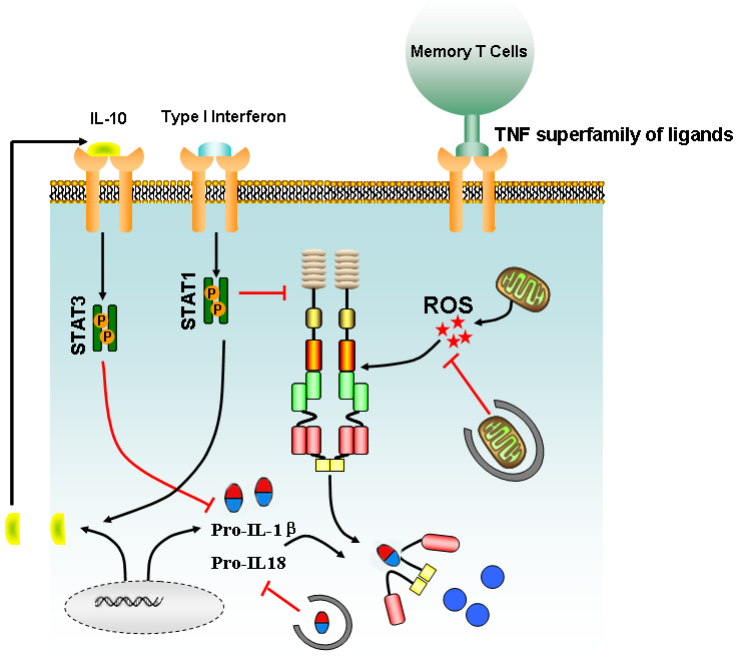
Main signaling involved in the regulation of inflammasome activation Type I interferon signaling triggers the production of IL-10, which in turn acts on cells in an autocrine or paracrine manner to suppress the intracellular concentration of pro-IL-1β *via* the stat3 pathway. ROS burst from damaged mitochondrion could drive activation of inflammasome, but autophagy could block the accelerated IL-1β/IL-18 production *via* degrading the damaged mitochondrion and sequestering intracellular stores of pro-IL-1β and IL-18. Meanwhile, effector and memory T cells could also suppress inflammasome activation *via* a cognate mechanism mediated by TNF superfamily and their receptors.

Similar to NF-κB, type I interferon is also important for inflammasome activation. AIM2 inflammasome activation following *F.tularensis* infection requires type I interferon stimuli, whereas macrophages deficient in the type I interferon secretion, result in reduced response of AIM2 inflammasome [43-45]. Although the precise mechanism of interferon signaling remains unclear, it has been proposed that type I interferon activates AIM2 inflammasome by generating cytosolic DNA from *F.tularensis* [44, 45]. However, type I interferon is also reported to inhibit inflammasome activation by two distinct mechanisms including the alteration of intracellular pro-IL-1β concentration and inhibiting caspase-1 activation [46]. The reduction of pro-IL-1β is determined by the capacity of type I interferon to induce the production of the anti-inflammatory cytokine, IL-10. IL-10 activation by STAT3 signaling pathway can inhibit the synthesis of pro-IL-1β and pro-IL-1α [47]. In addition, type I interferon is capable of suppressing caspase-1 activity *by* activation of the transcription factor, STAT1, subsequently inhibiting NLRP3 and NLRP1 inflammasome (Figure 3) [48]. Both *in vitro* and *in vivo* experiments further confirmed that IFN-β could suppress NLRP3 inflammasome, but the exact molecular mechanism that guides the preferential targeting of NLRP3 and NLRP1 inflammasome by type I interferon remains to be identified [49]. These data provide a duplex role of type I interferon in inflammasome modulation, which might be dependent on infected organisms or cell type and inflammation status. Besides the crosstalk between cytokines and inflammasomes, recent studies also suggest that the effector and memory T cells can block the activation of caspase-1 and IL-1β in macrophages and dendritic cells, mediated by CD40L, OX40L, and RANKL, which are all members of the TNF superfamily of ligands expressed on activated cells [50, 51]. Interestingly, although it is recognized that the T cells only target NLRP1 and NLRP3 inflammasomes, the underlying molecular mechanisms of how TNF ligands mediate the inhibition of caspase-1-dependent production of IL-1β is unknown and needs further investigation.

Evolutionarily, autophagy is a cell-protective mechanism against harmful stress that facilitates catabolic processes and inhibits anabolic metabolism. Growing evidence indicates that autophagy is a critical process participating in cancer initiation and metastasis, growth, and drug resistance [52, 53]. Intriguingly, recent reports have also indicated that autophagy regulates various aspects of the immune response, such as antigen presentation, cell death, and cytokine secretion in immune cells [54]. In autophagy-deficient *Atg16^−/−^* mice, it was observed that the levels of IL-1β and IL-18 were significantly elevated following LPS treatment. However, the elevated IL-1β and IL-18 expression was not due to enhanced transcriptional activity, but instead was attributed to over-activation of caspase-1 [55]. Subsequent mechanistic studies demonstrated that the augmented caspase-1 activity might be due to the failure of autophagy-deficient cells to clear damaged mitochondrion [55]. When autophagy is inhibited, excessive reactive oxygen species (ROS) will accumulate in the damaged mitochondrion, resulting in the release of mitochondrion DNA into the cytoplasm that finally triggers activation of NLRP3 inflammasome (Figure 3) [56, 57]. However, detailed mechanisms accounting for how mitochondrial ROS or mitochondrial DNA activates inflammasome are still unclear. Meanwhile, an inflammasome-independent mechanism of autophagy-mediated regulation of IL-1β expression was recently identified. Autophagosome could degrade pro-IL-1β, thereby restraining the substrate for caspase-1 processing (Figure 3) [58, 59]. Alternatively, autophagy inhibition could also activate the transcription of pro-IL-1β in human peripheral blood mononuclear cells [59]. Recently, autophagy was reported to affect inflammasome activity by influencing IL-1β translocation from the endoplasmic reticulum and Golgi apparatus [60]. Lastly, autophagy machinery is believed to participate in clearing large inflammasome complexes from cells in order to prevent excessive cell damage by IL-1β and IL-18 [61]. Therefore, the autophagic process regulates inflammasome activation at several levels.

## ROLE OF NOD-LIKE RECEPTORS IN CANCER DEVELOPMENT

The abnormal activation of inflammasome is linked to various types of human disease, such as cryopyrinopathies, gout, asbestosis, silicosis, Alzheimer's disease, and autoimmune diseases [62, 63]. To date, more than 70 inherited mutations have been identified associating with cryopyrinopathies occurrence, a large majority of which are situated within and around NLRP3 NACHT domain [64, 65]. These mutations are therefore believed to induce conformational changes that render NLRP3 constitutively active, resulting in continuous caspase-1 activation and release of IL-1β and IL-18 [65]. Besides, decreased NLRP3 expression and reduced IL-1β production have recently been linked with increased susceptibility to Crohn's disease in humans [66, 67]. Moreover, inflammasome deregulation was also recorded to contribute to the pathogenesis of experimental autoimmune encephalomyelitis [68]. Significantly, accumulating evidence also suggested that NLRs are closely correlated to cancer occurrence, but conflicting evidence also exists, which might be due to the dual functions of inflammasomes in promoting carcinogenic inflammation or eliminating malignant cells via the pyrotosis death pathway.

### NLRP3 signaling and its duplex role

**Figure 4:**
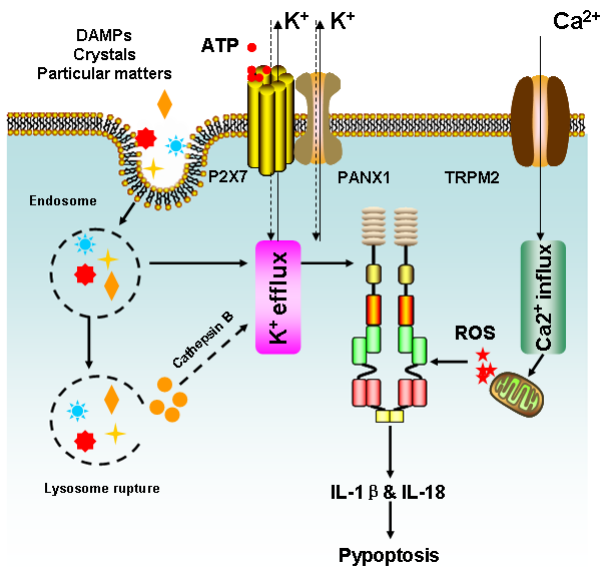
Simplified mechanisms for NLRP3 inflammasome activation Three distinct machineries have been proposed to account for NLRP3 activation, including K^+^ efflux, phagolysosomal destabilization and mitochondrial ROS burst. Extracellular ATP released from dying cells acts on purinergic receptor P2×7 and prompts pannexin-1 (PANX1) channels to enhance K^+^ efflux and result in NLRP3 activation. Meanwhile, PAMPs such as pore-forming toxins are also capable to facilitate K^+^ efflux and activate NLRP3 inflammasome. Besides, K^+^ efflux could be activated by crystals or particular maters, which enter the cells *via* endocytosis and trigger NLRP3 inflammasome *via* cathepsin B following lysosome rupture. Finally, intracellular Ca^2+^ accumulation could result in mitochondrion damage and lead to ROS burst, which may activate the NLRP3 inflammasome either directly or by inducing K^+^ efflux. Following NLRP3 activation, IL-1β and IL-18 will be greatly produced and results in inflammation or pyroptotic cell death.

NLRP3 is the most well-studied member of NLR family. It can be activated by a wide range of signals including infected pathogens, endogenous or environmental origins [69]. Based on current findings, 3 distinct mechanisms have been proposed to account for NLRP3 activation, including potassium efflux, phagolysosomal destabilization and mitochondrial ROS burst. Various bacterial pathogens can secret pore-forming toxins (e.g., nigericin from *Streptomyces hygroscopicus*, listeriolysin O from *Listeria monocytogenes*, pneumolysin from *Streptococcus pneumoniiae*, alpha-hemolysin from *Escherichia coli*) and subsequently activate the NLRP3 inflammasome by increasing potassium efflux [70-76]. In addition, bacterial and viral RNA is also reported to be an initial factor contributing to NLRP3 inflammasome assembly [77]. Moreover, extracellular ATP released from phagocytosed dying cells acts on purinergic receptor, P2×7 and induces pannexin-1 (PANX1) channels to promote potassium efflux and results in NLRP3 activation [78-80]. On the other hand, intracellular uptake of crystalline and particulate matters is also capable of causing lysosomal destabilization and release of cathepsin B, a sensor of NLRP3 [81-85]. Lysosome rupture-induced NLRP3 activation was also observed in cathepsin B-deficient cells, a phenomenon that may be attributed to potassium efflux (Figure 4) [86].

Notably, mitochondrial ROS generation is considered to be one of the most important mechanisms of NLRP3 activation. Pharmacological inhibition of mitochondrial ROS burst has been shown to prevent NLRP3-inflammasome formation [81, 82, 87]. Although the detailed molecular mechanisms of ROS-mediated NLRP3 activation remain largely unclear, calcium influx mediated by the transient receptor potential melastatin 2 (TRPM2) has been suggested to be a possible reason [87]. Extracellular calcium has been shown to activate the calcium sensing receptor (CASR) and thus lead to the release of calcium stores from the endoplasmic reticulum (ER), eventually triggering the formation of NLRP3 inflammasome (Figure 4) [88-90]. On the other hand, mitochondrial ROS burst is an upstream event leading to the loss of mitochondrial membrane potential, a pivotal event in inducing intrinsic apoptosis. Interestingly, overexpression of the anti-apoptotic protein, BCL2, was shown to limit the activation of NLRP3 inflammasome, indicating that apoptosis-regulated proteins might be closely correlated with NLRP3 activation [91, 92]. More recently, cIAP1, cIAP2, and XIAP have also been linked with inflammasome activation. cIAP1 and cIAP2 were found to enhance inflammasome activation by ubiquitinating and stabilizing caspase-1 and consequently, promoting Il-1β release, whereas concurrent inhibition of cIAP1, cIAP2, and XIAP was shown to limit caspase-1 activation [93-95]. Overall, these studies place the mitochondria as a potential player for inflammasome activation. However, the precise role of mitochondria in mediating NLRP3 inflammasome formation and subsequent promotion of carcinogenesis awaits clarification.

With regards to pro-tumorigenic ability, NLRP3 polymorphism is shown to be associated with melanoma susceptibility, colorectal cancer prognosis, and overall survival of myeloma [96]. In a Swedish case-control study, NLRP3 variant (rs35829419) was significantly more common in male patients than in controls (OR, 2.22; CI, 1.27-3.86) and showed strong association with nodular melanoma (OR, 2.89; CI: 1.33-6.30) [97]. It has been suggested that NLRP3 activation could suppress NK and T cell-mediated anti-tumor actions in a sarcoma mouse model and metastatic melanoma, whereas the population of myeloid-derived suppressor cells and Tregs were increased [98]. Consistently, NLRP3 silencing resulted in a 5-fold reduction in the number of tumor-associated myeloid-derived suppressor cells found in host mice, and NLRP3^−/−^ MDSCs were less efficient to reach the tumor site, demonstrating the critical role of NLRP3 in preventing cancer occurrence by modulating host immunity [99]. Furthermore, it was found that NLRP3-deficient mice generated less pulmonary metastasis in an orthotopic transplant mouse model of mammary adenocarcinoma [100]. In addition, chemotherapeutic agents, gemcitabine and 5-fluorouracil were shown to activate NLRP3-medaited inflammasome formation in myeloid-derived suppressor cells, leading to IL-1β production that is capable of inducing IL-17 secretion from CD4^+^ T cells and blunting the anticancer efficacy of chemotherapeutic drugs [101]. Accordingly, gemcitabine and 5-fluorouracil exert increased antitumor effects when tumors were established in NLRP3^−/−^ or Caspase-1^−/−^ mice, and NLRP3 activation by chemotherapeutic drugs is considered to be a positive regulator to promote cancer growth [101]. All these findings suggest the pro-tumorigenic role of NLRP3 in cancer development.

Although several lines of evidence have indicated the pro-carcinogenic activities of NLRP3, its role in cancer development remains controversial. NLRP3^−/−^ mice were shown to be more susceptible to cancer and the number of colon polyps in the AOM-DSS mouse model and the accelerated tumor growth in the carcinogenesis model was accompanied with drastically low levels of colonic IL-18, suggesting that NLRP3 may play a protective role against neoplasia formation and IL-18 might be closely associated with colon cancer initiation [102, 103]. Of note, IL-18 knockout mice generated more tumors than controls after administering AOM-DSS (azoxymethane- dextran sodium sulfate), whereas injection of recombinant IL-18 successfully restrained disease progression, which might be associated with MyD88-related pathway [104]. Similar anti-carcinogenic role of NLRP3 was also observed in hepatocellular carcinoma. Both mRNA and protein levels of NLRP3 were significantly down-regulated in the hepatic parenchymal cells derived from liver cancer biopsies compared to non-cancerous samples [105]. In this context, it is logical to deduce that NLRP3 may play a duplex role in controlling cancer growth. Thus, on one hand, NLRP3 could promote tumor cell survival through activation of NF-κB-stat1/3 pathway or by limiting cytotoxic immune cells infiltration, but on the other, it could suppress malignant progression by triggering mitochondrial apoptotic pathway or by enhancing immune-cytokine levels in the tumor microenvironment. In addition, the role of NLRP3 in cancer development might be tissue or cell dependent. For example, NLRP3 exhibits a protective role for colon cancer, but pro-carcinogenic effects for gastric and prostate malignancies [106]. Therefore, a much more comprehensive analysis of NLRP3 using conditional knockout models and pharmacological activators or inhibitors is needed to decode the precise effects of NLRP3 on cancer development in the future.

### Other NLRs in carcinogenesis

Besides NLRP3, a number of NLRs have also been shown to be associated with cancer progression. NLRC4 was identified as a downstream transcriptional target of p53, indicating the tumor suppressive role of NLRC4 [107]. Mice lacking NLRC4 had significantly increased tumor numbers and burden compared to the wild-type controls in the AOM-DSS colon cancer model, but no differences in inflammation severity were noted, implying that tumor regulation by NLRC4 might be mostly cell intrinsic and not through down-regulation of inflammation [108, 109]. Similar to NLRC4, both NLRP6 and NLRP12 were also found to play a critical role in AOM-DSS tumorigenesis. A significant increase in tumor number and burden was observed in NLRP6-deficient mice compared to wild-type controls after chemical induction. But unlike NLRC4, NLRP6-mediated protection against tumor formation is attributed to hematopoietic cells rather than intestinal epithelial cells, because similar numbers of tumor were observed between NLRP6 deficient mice and irradiated wild-type mice that were transplanted with NLRP6 deficient bone marrow [110-112]. By contrast, NLRP6 deficient mice that received wild-type bone marrow transplant were shown to have reduced tumorigenic ability, similar to that of wild-type animals [111]. In addition, genetic profiling of tumors from wild-type and NLRP6 deficient mice exhibited a significant increase in the number of genes in the Wnt and Notch signaling cascade from a set of 1,884 genes, supporting a novel role of NLRP6 in controlling intestinal proliferation [112]. Of note, proinflammatory cytokines such as TNFα and IL-6 were elevated in the tumor microenvironment, whereas the level of IL-18 was significantly reduced. Meanwhile, IL-18 silencing in NLRP6 deficient mice has been associated with increased colon cancer development, indicating the pivotal role of cytokines in mediating the anti-carcinogenic activities of NLRP6 [110]. Similar to NLRP6, NLRP12 was also considered to be a tumor suppressive molecule as shown in *ex vivo* and *in vivo* carcinogenic animal models. Mice lacking NLRP12 were found to be more susceptible to DSS-injury, accompanied by increased body weight loss, enhanced pathology scores coupled with severe inflammatory cell infiltration and high levels of cytokine production [113-115]. The AOM-DSS mouse model also revealed that NLRP12 deficient mice had accelerated colon tumor development and progression, which was demonstrated with over-activation of NF-κB signaling pathway and enhanced gene expression such as CXCL12 and CXCl13 [116, 117]. Taken together, the NLRP6/12-mediated protective mechanisms against tumorigenesis provide a complex network involving interactions between hematopoietic cells, cytokines, and epithelial cells and further show that experimental validation is needed to pinpoint the precise signaling transduction mode underlying their anti-carcinogenic effects.

### Double-edged swords of pyroptosis

Pyroptosis is a critical self-protection mechanism responding to pathogen invasion by inducing pro-inflammatory cell death. Unlike apoptosis, pyroptosis is characterized by cytoplasmic swelling and early cellular membrane rupture, which happens following caspase activation, nuclear condensation and DNA fragmentation [118]. Although the precise mechanisms underlying pyroptosis induction still remains elusive, the products released from dead cells may limit malignant cell survival and proliferation by activating the innate immune response. Increasing evidence validates that dying tumor cells following chemotherapy might activate the NLRP3 inflammasome of dendritic cells *via* P2×7 purinergic receptors, thus priming tumor-specific interferon-γ-producing T lymphocytes to limit cancer growth [119]. Moreover, mice lacking P2×7 or NLRP3 failed to prime interferon-γ-producing CD8^+^ T cells after chemotherapy, and anthracycline-treated breast cancer patients with P2×7 mutation developed metastatic lesions more rapidly than normal individuals [100]. Notably, a novel therapeutic strategy is in development to foster dendritic cells-mediated anti-tumor immunity *via* acceleration of pyroptosis of cancer cells by oncolytic viruses [120]. However, several studies have also indicated that pyroptosis might contribute to tumorigenic ability after inflammasome activation [121, 122]. These conflicting findings may be attributed to differences in the redox status of model cells and specific molecules involved in the process. For example, the reduced form of HMGB1 released from dying cells could trigger dendritic cells to induce anti-tumor immune response, while the oxidized form of HMGB1 would be unable to activate the immune response [123, 124]. In addition, the role of pyroptosis in cancer development might critically depend on the cell type. Pyroptosis of immune cells might bring harmful consequences to tumor immunoediting, while cancer cell pyroptosis would improve anti-cancer immunity. Overall, impaired pyroptosis has been considered to be a potential mechanism linking chronic inflammation to cancer initiation, and pyroptosis targeting is becoming a novel strategy to prevent cancer and improve cancer therapeutic efficacy.

## CONCLUSIONS

Remarkable advancements in recent years have greatly increased our understanding of NLRs function and the associated inflammasome in host defense and disease pathogenesis. NLR containing inflammasomes are not only important for fighting against bacterial, fungi and viruses, but also appear to be a critical step in mediating cancer initiation and progression. Inflammasome activation would create a pro-inflammatory microenvironment for inducing malignant transformation, and suppress local immunity caused by NK or T cells. In addition, chemotherapeutic agents were found to activate inflammasome defense, which had positive feedback to support cancer growth. Notably, inflammasome-related autophagy is also believed to significantly contribute to cancer drug resistance and metastasis. All these findings greatly highlight the role of inflammasome as a novel target to prevent and treat cancer.

Despite mounting evidence listed above suggesting the potential of the inflammasome as a promising marker for cancer prevention, contrary data also exists to imply that inflammasome signaling could behave as a kind of anti-cancer mechanism. Mice lacking NLRP3 or NLRC4 show higher susceptibility to colon cancer following AOM-DSS treatment, and aberrant inflammasome formation leads to inhibition of tumor suppressor genes such as p53 and over-activation of oncogenes such as Wnt. What's more, inflammasome-mediated pyroptosis is also considered to play a critical role in recruiting dendritic cells to limit cancer growth. Based on the conflicting evidence, a number of questions remain unanswered. Whether or not inflammasome-related carcinogenesis is cell dependent is an important question. A second question is whether a specific NLR would exhibit different bioactivity correlating with a cancer stage. Meanwhile, there are 22 NLR members in humans and it is unknown how these NLR molecules are activated or how they interact with each other. The complex network is awaits elucidation. There is also a lot of interest to identify novel ligand-receptor binding molecules, novel signaling pathway and novel targets for cancer prevention or therapy. The inflammasome is becoming a significant research topic in tumor microenvironment field and there is every likelihood that it could be developed as important biomarker for cancer diagnosis or prognosis prediction. Meanwhile, drug discovery targeting inflammasome modulation is also expected to improve cancer therapeutic efficacy to successfully reduce cancer risk. Taken together and given the emerging role of inflammasome in cancer development, understanding its signaling network and pathological significance might bring novel strategies for malignancy therapy and prevention.
